# Examining the decline in modern contraception usage among married women in Bangladesh: Applying Blinder-Oaxaca decomposition analysis

**DOI:** 10.1371/journal.pone.0304122

**Published:** 2024-05-23

**Authors:** Samia Kabir, Muhammad Tareq, Md. Ismail Hossain

**Affiliations:** 1 Department of Statistics, Jagannath University, Dhaka, Bangladesh; 2 Department of Mathematics and Natural Sciences, BRAC University, Dhaka, Bangladesh; ICMR - National Institute for Research in Reproductive and Child Health (ICMR-NIRRCH), INDIA

## Abstract

**Objectives:**

Controlling population expansion and reducing unintended pregnancies through the use of modern contraceptives is a cost-effective strategy. In recent years, the rate of modern contraceptive use in Bangladesh has been declining. So, this study aimed to investigate the associated factors of the deterioration in modern contraceptive usage.

**Methods:**

This study used data from two successive Bangladesh Demographic and Health Surveys (2014 and 2017–18) and applied the Blinder-Oaxaca decomposition analysis to understand the drivers. A popular binary logistic regression model is fitted to determine the factors that influence the use of modern contraceptive methods over the years.

**Results:**

This study revealed that highly educated women were more likely to use modern contraception methods, and their use increased by 3 percent over the years. Factors such as women’s working status, husband’s education, number of living children, and fertility preference were found significantly associated with decreased usage of modern contraception methods over years. The result of the Blinder-Oaxaca (BO) decomposition analysis found a significant decrease between 2014 and 2018. Respondent’s age, working status, husband’s age, opinion on decision making, region, and media exposure were the most significant contributors to explaining the shift between 2014 and 2018. The two factors that contributed most to narrowing the difference between the two surveys were women’s decision on own health (26%), and employment status (35%).

**Conclusions:**

The factors that influence modern contraceptive prevalence are important to know for policy implication purposes in Bangladesh. The findings indicate the need for further improvement of factors for balancing the usage of modern contraception methods.

## 1. Introduction

High fertility and therefore high population growth rates are one of the major economic and social problems in developing countries [1]. The use of contraception plays a significant role in fertility and maternal mortality reduction. The use of contraceptive methods introduced various family planning programs to reduce the impact of abortion, miscarriage, stillbirth and at the same time increase the impact of management and postpartum care outcomes. In Bangladesh, it has been found that mothers under the age of 18 have a higher risk of death during childbirth because most women are not physically or mentally ready, and subsequent complications of various pregnancies increase their risk. The only way to prevent complications and death during pregnancy is to arrange proper family planning, and the use of contraceptives is a significant component of family planning [2–4]. Contraception is the use of various devices, agents, drugs, sexual practices or surgical procedures during any contraceptive, or sexual activity that is intentionally used to prevent a woman’s pregnancy. Contraceptive methods are divided into two types, Modern contraceptives and Traditional contraceptives method. Modern contraceptive methods comprise female sterilization, male sterilization, pills, depot implants, male condoms, female condoms, Intra Uterine Devices (IUDs), Lactational Amenorrhea Methods (LAM), and emergence contraception [5,6]. The use of modern contraceptives is currently recognized as the most cost-effective approach to improving general reproductive health and global socio-economic development [5].

Bangladesh is a densely populated country in South Asia with a population of 140 million and 900 inhabitants per square kilometer [1,7]. This population of the country needs to control the explosion which depends on the increased use of birth control methods [1]. Bangladesh has made significant progress in the use of contraception in past four decades [8]. There are several studies which have found significant factors associated with the use of modern contraceptives, such as respondent’s age [1,3,9,10], education [7,3,11,12], region [5,13,14], fertility preferences [15], women’s decisions on own health care [16,17], and the number of surviving [9,18–20]. Despite previous Bangladesh Demographic and Health Surveys (BDHSs) indicating a significant upward trend in modern contraceptive usage (from 2007 to 2014) [21–23], according to recent Bangladesh Demographic and Health Survey reports, the prevalence rate of modern contraception suddenly declined and engendered a gap from the previous year [23,24]. However, there was no clear idea about the reasons behind this change in using modern contraception methods between 2014 and 2017–18. Based on this research gap, the main objective of this study is to investigate drivers of the gap in the modern contraception usage.

## 2. Materials and methods

### 2.1 Data source

The analysis is based on secondary data, which was extracted from Bangladesh Demographic and Health Survey (BDHS) of 2014 and 2017–18. These surveys were implemented under the authority of the National Institute of Population Research and Training (NIPORT), and Mitra Associations. The BDHS 2014 and BDHS 2017–18 datasets share several key similarities, making them comparable for the study of modern contraceptive methods. Both surveys employ a nationally representative sample design, utilizing a stratified two-stage cluster sampling technique. They target women of reproductive age through face-to-face interviews conducted by trained interviewers. As such, the sample size, respondent types, data collection modes, and source of information are consistent between the two datasets.

### 2.2 Sample design & sample size

The BDHS surveys in 2014 and 2017–18 utilized a two-stage stratified cluster sampling method to ensure the representativeness of the data. In the first stage, clusters were selected with probability proportional to size from enumeration areas across Bangladesh. The number of clusters selected increased slightly from 600 in 2014 to 675 in 2017–18. Each selected cluster was further sampled using systematic sampling to obtain approximately thirty households per cluster, resulting in a robust sample size for reliable estimates of demographic and health variables nationwide. In both survey rounds, the women’s questionnaire was administered to households covering substantial number of households and ever-married women aged reproductive age (15 to 49 years). BDHS covered 17,989 and 20,160 households in 2014 and 2017–18 data sets respectively. From these sample households 17,863 and 20,127 ever-married women from 15–49 age were interviewed. In this study, data are restricted to currently married women aged 15–49. Based on the criteria, we obtained 16,858 and 18,984 currently married women from 2014 and 2017–18. For our convenience in interpretation, we use BDHS 2017–18 as BDHS 2018.

### 2.3 Dependent variable

The dependent variable for this study is the use of modern contraceptive methods. It indicates respondents’ current use of any method of contraception at the time of the survey. For the purpose of classification, we assigned the label "Yes" to respondents who reported current usage of modern contraceptive methods, and the label "No" to those utilizing other methods or not using any contraceptive method.

### 2.4 Independent variables

This study deals with a large number of independent variables. The variables are: respondent’s age, education, currently working status, husband’s age, education, wealth status, number of living children, fertility preference, women’s decisions on own health care, religion, region, residence, and media exposure.

### 2.5 Statistical analysis

#### 2.5.1 Univariate & bivariate analysis

An overview of the variables was conducted through frequency distribution. We performed bivariate distributions to present the summary of the independent variables. To explore the association between dependent and independent variables, *χ*^2^ test was performed. Mathematically, the chi-square statistics has been defined as,

$$\chi^{2}=\sum_{i=1}^{n}\frac{{\left({Observed\;frequency}_{i}-{Expected\;frequency}_{i}\right)}^{2}}{{Expected\;frequency}_{i}}$$

This statistic follows a chi-square distribution with (*Number of row*−1)×(*Number of column*−1) degrees of freedom.

#### 2.5.2 Multivariable analysis

Multivariable analysis involved conducting separate binary logistic regression analyses for the years 2014 and 2018 to identify factors associated with the dependent variable: use of modern contraceptive methods. Independent variables were included in each logistic regression model to evaluate their influence on the likelihood of the dependent variable. A significance level of 95% was utilized, and adjusted odds ratios (ORs) along with their corresponding 95% confidence intervals (CIs) were computed to quantify the magnitude and direction of associations.

Let *Y*_*i*_ denote the binary outcome variable for the *i*^*th*^ observation, here,

$$Y_{i}=\left\{ \begin{array}{c} 1,if\;respondent\;is\;user\;of\;modern\;contraception\;method\;\\ 0,if\;respondent\;is\;not\;user\;of\;modern\;contraception\;method\; \end{array}\right.$$

*X*_*i*1_,…,*X*_*ik*_ be a set of explanatory variables i.e., *i*^*th*^ observation is explained by *k* independent variables and *π*_*i*_ is the parameter of Bernoulli distribution of outcome variable.

In this study, *i* typically ranges from 1 *to n*, representing the total number of observations or units in our sampled data set. Each *i* corresponds to a specific observation or unit. Additionally, *k* represents the number of independent variables with each observation. For our study, the value of *i* for BDHS-2014 was 16,858, and for BDHS 2018, it was 18,984. Both surveys had *k* = 13 independent variables. In general, for the binary logistic regression model, the probability of success of dependent on independent variables can be written as,

$$P(Y_{i}=1)=\pi_{i}=\frac{exp(\beta_{0}+\beta_{1}X_{i1}+\cdots +\beta_{k}X_{ik})}{1+exp(\beta_{0}+\beta_{1}X_{i1}+\cdots +\beta_{k}X_{ik})}$$

The strategy is to apply log odds to the response and fit the model. The above ratio can be expressed as,

$$g(X)=logit(\pi_{i})=log\frac{\pi_{i}}{1-\pi_{i}}=\beta_{0}+\beta_{1}X_{i1}+\cdots +\beta_{k}X_{ik}$$

The result was presented as odds ratio (OR) and 95% confidence interval (CI).

#### 2.5.3 Decomposition analysis

We analyzed changes in modern contraception use between 2014 and 2018 in Bangladesh using Blinder-Oaxaca (BO) decomposition analysis. This method breaks down changes into "endowments," "coefficients," and "interactions" components. Endowments represent differences in explanatory variables, while coefficients indicate changes due to determinants.

Let *Y*_*i*_ be the dependent variable, is affected by a single independent variable *X*_*i*_, and we have two groups, survey 2014 and survey 2018 where *β* be the coefficients to be estimated. Then, the use of modern contraception for the 2014 and 2018 women are given by following equations,

$$Y_{i}^{2014}=\beta^{2014}X_{i}+{ℇ}_{i}^{2014}$$

$$Y_{i}^{2018}=\beta^{2018}X_{i}+{ℇ}_{i}^{2018}$$

Thus, the survey year shift in the mean use of modern contraception Δ*Y* = (*Y*^2014^−*Y*^2018^) can be written as,

$$(Y^{2014}-Y^{2018})=\beta^{2014}X^{2014}-\beta^{2018}X^{2018}$$

Where, *X*^2014^ and *X*^2018^ are the independent variable at their means for the survey year 2014 and 2018. The overall year 2014–2018 gap could be decomposed into a gap that attributable to difference in the level of covariates, *X*′*s*, and a gap that is attributable to difference on coefficients, *β′s* as written,

$$\begin{array}{l} \Delta Y=(X^{2014}-X^{2018})\beta^{2018}+(\beta^{2014}-\beta^{2018})X^{2014}+(X^{2014}-X^{2018})(\beta^{2014}-\beta^{2018})\\ =\Delta X\beta^{2014}+\Delta \beta X^{2018}+\Delta X\Delta \beta \\ =E+C+CE \end{array}$$

Here the overall shift in using modern contraception methods between two surveys is comprised of the shift in endowments effect (E), coefficients effect (C), and the interactions effect (CE).

**Fig 1:**
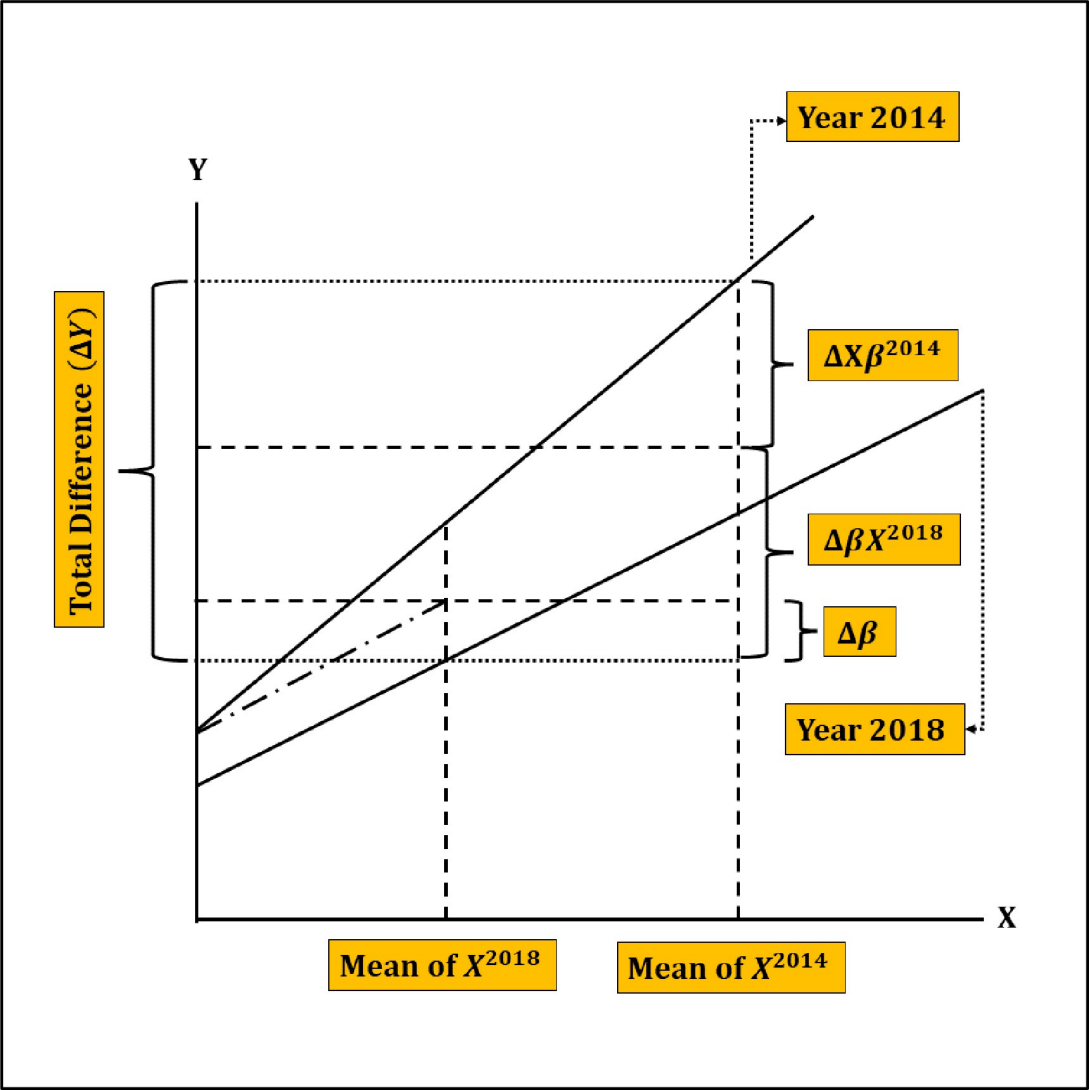
A graphical representation of the Blinder-Oaxaca decomposition.

In Fig 1, a simple visual of Blinder-Oaxaca decomposition is presented. It illustrates modern contraception usage (*Y*) as the dependent variable, correlated positively with a single predictor variable *X*. Notably, the mean/proportion level of *X* is higher in 2014 compared to 2018. Furthermore, differences in intercepts and slopes are observed between the survey years 2014 and 2018.

### 2.6 Software

For data management the Statistical Package for Social Sciences (SPSS) version 25 used. The univariate, bivariate, and multivariate analysis were performed by SPSS. STATA version 16 was used to perform the inequality and decomposition analysis in this study.

## 3. Result

### 3.1 Socio-demographic characteristics

Table 1 displays the distributions of the selected independent variables from BDHS 2014 and 2018. The highest percentage of women across the study period were aged 25–35 years. The result showed that the percentage of women with secondary and higher education increased from 47.4% in 2014 to 53.4% in 2018. The percentage of women’s working status increased from 31.9% to 47.0% over the years. Primary level education among husbands increased from 27.8% to 32%. More than 53% of women had 1–2 living children over the study periods. A large portion of women showed aversion toward having another child. Women’s opinions on making any decision increased from 47.2% in 2014 to 59.4% in 2018. From each sample, approximately 34.7% of women lived in the central region. More than 70% of women were from rural areas. Women’s exposure to media decreased from 46.8% in 2014 to 44.0% in 2018.

**Table 1:** Distribution of married women aged 15–49 by socio-demographic characteristics in 2014 and 2018.

Variables	BDHS 2014		BDHS 2018	
	n = 16858	%	n = 18984	%
**Women’s age (in years)**				
15–24	5150	30.6	5441	28.7
25–34	6168	36.6	6753	35.6
35–49	5539	32.9	6790	35.8
**Women’s education**				
No education	3949	23.4	2947	15.5
Primary	4916	29.2	5904	31.1
Secondary & above	7993	47.4	10133	53.4
**Women’s working status**				
Working	5371	31.9	8921	47.0
Not working	11486	68.1	10064	53.0
**Husband’s age (in years)**				
15–24	905	5.4	972	5.1
25–34	5097	30.2	5446	28.7
35+	10855	64.4	12566	66.2
**Husband’s education**				
No education	4715	28.0	4131	21.8
Primary	4680	27.8	6080	32.0
Secondary & above	7463	44.3	8773	46.2
**Wealth Status**				
Poor	6320	37.5	7203	37.9
Middle	3394	20.1	3846	20.3
Rich	7143	42.4	7936	41.8
**Number of living children**				
0	1707	10.1	1938	10.4
1–2	8948	53.1	10196	53.7
3 and more	6203	36.8	6810	35.9
**Fertility preference**				
Want more	5293	31.4	6339	33.4
Don’t more	11564	68.6	12645	66.6
**Decision on own health**				
None	3632	21.5	2263	11.9
At least one	2579	15.3	2435	12.8
At least two	2692	16.0	3016	15.9
All decision	7955	47.2	11270	59.4
**Religion**				
Islam	15187	90.1	17205	90.6
Others	1670	9.9	1779	9.4
**Region**				
Northern	3953	23.4	4892	25.8
Southern	2779	16.4	3261	17.2
Central	5857	34.7	6332	33.4
Eastern	4269	25.3	4498	23.7
**Residence**				
Urban	4709	27.9	5378	28.3
Rural	12149	72.1	13607	71.7
**Media exposure**				
Exposed	8961	46.8	8351	44.0
Not exposed	7897	53.2	10633	56.0
**Use of modern contraception**				
Yes	9112	54.1	9853	51.9
No	7745	45.9	9131	48.1

### 3.2 Association between use of modern contraception and selected variables

Table 2 shows the bivariate association between contraception method user categories and selected independent variables. The use of modern contraceptive methods decreased by 4% among 25-34-year-old women from 2014 to 2018. The study found that educated women were less likely to use modern contraceptive methods over the years. More than fifty percent of husbands with 35 or more years of age were found to be interested in using modern contraception methods. The percentage of husbands using modern contraceptive methods decreased (from 52.7% to 49.4%) among those with secondary and higher education.

**Table 2:** Bivariate relationship between use of modern contraception and socio-demographic characteristics in 2014 and 2018.

Variables	Use of modern contraception			
	BDHS 2014		BDHS 2018	
	Yes (%)	No (%)	Yes (%)	No (%)
**Women’s age (in years)**				
15–24	51.5	48.5	48.2	51.8
25–34	63.7	36.3	59.7	40.3
35–49	45.7	54.3	47.1	52.9
** p value**	<0.001		<0.001	
**Women’s education**				
No education	50.5	49.5	48.7	51.3
Primary	55.2	44.8	53.7	46.3
Secondary & above	55.1	44.9	51.8	48.2
** p value**	<0.001		<0.001	
**Women’s working status**				
Working	58.8	41.2	57.0	43.0
Not working	51.8	48.2	47.4	52.6
** p value**	<0.001		<0.001	
**Husband’s age (in years)**				
15–24	46.7	53.3	46.9	53.1
25–34	56.0	44.0	51.6	48.4
35+	53.8	46.2	52.4	47.6
** p value**	<0.001		0.003	
**Husband’s education**				
No education	54.5	45.5	53.7	46.3
Primary	55.8	44.2	54.2	45.8
Secondary & above	52.7	47.3	49.4	50.6
** p value**	0.003		<0.001	
**Wealth Status**				
Poor	55.2	44.8	54.8	45.2
Middle	54.5	45.5	50.2	49.8
Rich	52.9	47.1	50.1	49.9
** p value**	0.024		<0.001	
**Number of living children**				
0	22.6	77.4	20.8	79.2
1–2	58.9	41.1	56.0	44.0
3 and more	55.7	44.3	54.7	45.3
** p value**	<0.001		<0.001	
**Fertility preference**				
Want more	46.8	53.2	43.3	56.7
Don’t more	57.4	42.6	56.2	43.8
** p value**	<0.001		<0.001	
**Decision on own health**				
None	47.9	52.1	46.9	53.1
At least one	54.7	45.3	48.5	51.5
At least two	56.3	43.7	52.7	47.3
All decision	55.9	44.1	53.4	46.6
** p value**	<0.001		<0.001	
**Religion**				
Islam	53.8	46.2	51.2	48.8
Others	56.6	43.4	59.1	40.9
** p value**	0.031		<0.001	
**Region**				
Northern	61.9	38.1	56.8	43.2
Southern	55.7	44.3	51.8	48.2
Central	54.2	44.8	53.2	46.8
Eastern	45.5	54.5	44.8	55.2
** p value**	<0.001		<0.001	
**Residence**				
Urban	56.2	43.8	54.9	45.1
Rural	53.2	46.8	50.7	49.3
** p value**	<0.001		<0.001	
**Media exposure**				
Exposed	55.2	44.8	52.7	47.2
Not exposed	52.8	47.2	50.7	49.3
** p value**	0.002		0.004	

The study found a decreased rate of using modern contraceptive methods among middle-class women over the years (from 54.5% to 50.2%). Women with 1–2 living children were interested in using modern contraceptive methods. Over 56% of modern contraceptive users were found to be reluctant to have more children. Women’s opinions on making the decision have dwindled over time. The percentage of women using modern contraceptive methods decreased among those who resided in the Northern (from 61.9% to 56.8%) and Southern (from 55.7% to 51.8%) regions from 2014 to 2018. Modern contraceptive users were mostly from urban areas. The percentage of women using modern contraceptive methods increased from 44.8% to 47.2% among women who were exposed to the media.

### 3.3 Factors affect in using modern contraception methods over the years

The binary logistic model is fitted to identify the factors that affect the use of modern contraceptive methods in 2014 and 2018 and the results are presented in Table 3. Women aged 35–49 were less interested in using modern contraceptive methods compared to 15–24 aged women. The use of modern contraception methods increased by 3% among women from secondary and above education over the years. The result showed that unemployed women were 30% (2014) more likely to use modern contraceptives compared with employed women, which decreased by 26% in years 2018. Husband’s modern contraception use increased with age 25–34 years compared to 15–24 over years (OR = 0.72 in 2014, OR = 0.60 in 2018). The odds of using modern contraception methods were high among women who had 3 or more children (OR = 7.58 in 2014, OR = 6.68 in 2018) than women who had no children. About 67% of women who preferred no children used modern contraceptive methods in 2018. Women’s opinions on making any decision were decreased over the year. Women from other religions were 1.45 (95% CI: 1.31, 1.61) times more likely to use modern contraceptive methods in 2018, which was 1.18 (95% CI: 1.05, 1.31) times in 2014. In 2018, rural women showed a 25% lower interest in modern contraceptive methods compared to urban women, while in 2014, this figure was 21% lower for rural women.

**Table 3:** Results of fitting binary logistic models to identify the factors affecting use of modern contraceptive methods over the years.

Variable	BDHS 2014	BDHS 2018
	OR [95% CI]	OR [95% CI]
**Women’s age (in years)**		
15–24 (Ref.)	1	1
25–34	0.99 [0.89, 1.10]	0.90 [0.81, 0.99]
35–49	0.40 [0.35, 0.46] ***	0.45 [0.39, 0.51] ***
**Women’s education**		
No education (Ref.)	1	1
Primary	1.14 [1.04, 1.25] **	1.18 [1.07, 1.30] **
Secondary & above	1.30 [1.17, 1.45] ***	1.33 [1.19, 1.48] ***
**Women’s working status**		
Working (Ref.)	1	1
Not working	1.30 [1.17, 1.45] ***	0.74 [0.69, 0.79] ***
**Husband’s age (in years)**		
15–24 (Ref.)	1	1
25–34	0.72 [0.61, 0.85] ***	0.60 [0.51, 0.71] ***
35+	0.58 [0.48, 0.70] ***	0.52 [0.43, 0.62] ***
**Husband’s education**		
No education (Ref.)	1	1
Primary	0.99 [0.90, 1.08]	0.96 [0.82, 1.05]
Secondary & above	0.92 [0.83, 1.01]	0.87 [0.89, 0.95] **
**Wealth Status**		
Poor (Ref.)	1	1
Middle	0.97 [0.89, 1.07]	0.86 [0.79, 0.94] **
Rich	0.91 [0.82, 1.00]	0.84 [0.77, 0.92] ***
**Number of living children**		
0 (Ref.)	1	1
1–2	5.37 [4.68, 6.17] ***	5.13 [4.49, 5.85] ***
3 and more	7.58 [6.44, 8.92] ***	6.68 [7.73, 7.80] ***
**Fertility preference**		
Want more (Ref.)	1	1
Don’t more	1.49 [1.37, 1.64] ***	1.67 [1.53, 1.82] ***
**Decision on own health**		
None (Ref.)	1	1
At least one	1.28 [1.15, 1.43] ***	0.98 [0.88, 1.12]
At least two	1.22 [1.09, 1.35] ***	1.09 [0.97, 1.23]
All decision	1.23 [1.31, 1.34] ***	1.03 [0.94, 1.14]
**Religion**		
Islam (Ref.)	1	1
Others	1.18 [1.05, 1.31] **	1.45 [1.31, 1.61] ***
**Region**		
Northern (Ref.)	1	1
Southern	0.49 [0.44, 0.54] ***	0.64 [0.59, 0.70] ***
Central	0.72 [0.66, 0.78] ***	0.91 [0.84, 0.99] *
Eastern	0.81 [0.73, 0.90] ***	0.85 [0.77, 0.93] ***
**Residence**		
Urban (Ref.)	1	1
Rural	0.79 [0.74, 0.87] ***	0.75 [0.69, 0.81] ***
**Media exposure**		
Exposed (Ref.)	1	1
Not exposed	1.16 [1.07, 1.26] ***	1.20 [1.12, 1.29] ***

Ref. = Reference Category, Significant at ***P<0.001

**P<0.01

*P<0.05.

### 3.4 Decomposition of the usage between 2014 and 2018 shift

Table 4 shows the summary of Blinder-Oaxaca decomposition analysis. As shown in the Table, the probability of using modern contraceptive methods was 0.519 (95% CI: 0.511, 0.527, p <0.001) in 2018, which was 0.540 (95% CI: 0.531, 0.551, p <0.001) in 2014. There was a significant decreasing pattern in using modern contraceptive methods between two surveys and the average difference was 0.022 (95% CI: 0.009, 0.034, p <0.01). This difference is decomposed into endowments, coefficients, and interactions. The result showed that endowments and coefficients were significantly affect the usage of modern contraceptive methods between 2014 and 2018. About 41% (-0.009 units from the total of 0.022 units, p <0.01) of endowments explain the differences between 2014 and 2018. This means that these differences in respondents’ endowments explain 41 percent of the discrepancies observed between the two Bangladesh Demographic and Health Surveys (BDHSs). On the other hand, the average difference accounted for coefficients was 0.029.

**Table 4:** Blinder-Oaxaca decomposition estimates of the usage of modern contraceptive methods among 15–49 aged married women between 2014 and 2018.

	Estimate [95% CI]
Predicted modern contraceptive usage for BDHS 2014	0.541 [0.531, 0.551] ***
Predicted modern contraceptive usage for BDHS 2018	0.519 [0.511, 0.527] ***
Difference	0.022 [0.009, 0.034] **
Difference due to endowments	-0.009 [-0.016, -0.003] **
Difference due to coefficients	0.029 [0.017, 0.043] ***
Difference due to interactions	0.001 [-0.005, 0.008]

Significant at ***P<0.001

**P<0.01

*P<0.05.

### 3.5 Contribution of determinant in the shift between 2014 and 2018

The contribution of endowments and coefficients effect for explaining the shift in using modern contraceptive methods between two surveys (2014 and 2018) was presented in Table 4. The result found that respondent’s age, working status, husband’s age, opinion on making any decision, region, and media exposure were the most significant contributors to explaining the shift between 2014 and 2018. A negative contribution indicates that the determinant was narrowing the shift between 2014 and 2018, and vice-versa. Table 5 shows that 35–49 aged women (27%) and 35+ aged husbands (10%) were the highest contributors to widening the shift between 2014 and 2018. The shift that was accounted for coefficient effects, for example, women’s opinions on at least one (40%) and all decision (85%) have contributed a lot to maximize the gap. Unemployed women (35%) and women’s opinions on all decision (26%) has contributed to narrowing the shift between 2014 and 2018. Women’s higher education contributed about 16% for diminishing the shift between two surveys. In addition, women from eastern region (12%) and unexposed to media (4%) has contributed a lot to lessen the shift.

**Table 5:** Covariates contribution in the shift between 2014 and 2018 among 15–49 aged married women.

Variables	Endowments contribution (%)	Coefficient contribution (%)
**Women’s age (in years)**		
15–24	Ref.	Ref.
25–34	-0.12	35.20
35–49	27.74***	-39.08
**Women’s education**		
No education	Ref.	Ref.
Primary	-2.65	-10.46
Secondary & above	-16.39**	-11.69
**Women’s working status**		
Working	Ref.	Ref.
Not working	-34.95***	52.99
**Husband’s age (in years)**		
15–24	Ref.	Ref.
25–34	-5.40*	55.31
35+	10.15**	81.65
**Husband’s education**		
No education	Ref.	Ref.
Primary	0.57	7.23
Secondary & above	1.79	26.04
**Wealth Status**		
Poor	Ref.	Ref.
Middle	0.03	26.52
Rich	-0.59	33.97
**Number of living children**		
0	Ref.	Ref.
1–2	-11.09	26.06
3 and more	19.64	47.64
**Fertility preference**		
Want more	Ref.	Ref.
Don’t more	8.43**	-76.76
**Decision on own health**		
None	Ref.	Ref.
At least one	6.40**	40.81**
At least two	0.17	17.68
All decision	-26.56***	85.87**
**Religion**		
Islam	Ref.	Ref.
Others	0.91	-21.62**
**Region**		
Northern	Ref.	Ref.
Eastern	-12.23**	-71.73***
Central	-4.83	-87.36**
Southern	1.51	-7.09
**Residence**		
Urban	Ref.	Ref.
Rural	-0.94	45.23
**Media exposure**		
Exposed	Ref.	Ref.
Not Exposed	-4.51**	-18.23

Ref. = Reference Category, Significant at ***P<0.001; **P<0.01; *P<0.05.

## 4. Discussion

Although Bangladesh has made progress in increasing the rate of using modern contraception among married women, the sudden decrease in 2018 was engendered a challenge. Our study investigated the percentage and associated factors of the modern contraceptive usage among married women in Bangladesh by applying binary logistic regression model. In this study, we comprehensively examine the underlying demographic and socioeconomic factors that explain two consecutive surveys disparities in modern contraceptive usage among married women by introducing the Blinder-Oaxaca decomposition method.

According to the results of this study, the rate of modern contraception usage Bangladesh was 51.9% in 2018, which was lower than the estimate of 2014 (approximately 54%). Though the prevalence of modern contraceptive usage was decline in Bangladesh, this percentage was higher than Ghana [25].

This study assesses the determinants of using modern contraception methods and the contribution of determinants for shifting the change of using modern contraception methods between 2014 and 2018. The result found significant association between respondent’s age and the use of modern contraception methods over the years. We found that younger women used more modern contraception methods than older women. A recent study conducted by Sidibé revealed that women with younger age (20–24) became independent and more likely to decide about the utilization of modern contraceptive methods [26]. The odds of using modern contraception methods among women aged 35–49 was increased in 2018 compared to previous year.

Education is considered as important factor of using contraception methods. The result showed that educated women were likely to use modern contraceptive methods and it increased over the years. The reason why educated women use more modern contraceptives may be because they are more aware of the use of contraceptives and have sufficient knowledge and cognitive skills about methods [7]. Women who had no education were less interested in using modern contraceptive methods which is similar to other studies [27,28]. Women with secondary and above education significantly affects the usage of modern contraception methods in our study. Previous findings concluded that women with secondary and higher education used contraception methods compared to uneducated [3,29,30].

The study resulted that respondent’s working status significantly affects the use of modern contraception methods. Employed women chose to use modern contraceptive methods in 2018, which was higher than the previous year. It may be that working status empowers women financially, gives access to contraceptive and health care information, and this empowerment encourages the use of contraceptives [18]. Different researchers from past studies found a significant association with women’s working status [17,18]. Husband’s age, educational attainment, and wealth status also identified as significant factors in using modern contraceptive methods in 2014 and 2018.

The odds of using modern contraception methods among women who were unwilling have children increased over the years. The study found that women who preferred no more children were more likely to use modern contraceptive methods compared to preferred one. The tendency of use of contraception among women who want no more children indicates that a growing percentage of women want to use modern contraceptives, effective and long-term contraceptives more easily obtainable which helps them to attain their fertility goals [15].

The study found significant relationship between women’s opinions on making any decision and the use of modern contraception methods in 2014. The result concluded that women’s opinion on making any decision has been decreasing between the years. Previous studies suggested that higher education, and employment status could be improved women’s autonomy in decision making on health care [17,31,32]. We found this rate was decreased in latter year.

Women who resided in urban areas used more modern contraception methods than rural women. Some previous researchers found in their study that the use of modern contraceptives has declined among women in rural areas. According to various studies, the main reason for this decline is the lack of accessibility and affordability of family planning services in rural areas [5,13,14].

The role of the media is immense in raising awareness and knowledge of contraceptive use, and even dispelling misinformation about family methods [33–35]. The study showed significant association between media exposure and the use of modern contraception methods. The rate of using modern contraception methods were high among women who remained unexposed to media and it increased over the years. Though this finding is not in line with the previous studies, we consider media exposure as an important factor of using contraception methods. Previous researchers concluded that information and benefits of using modern contraceptives are coming from the media. If we can increase the number of media exposures, then the number of modern contraceptive users will be increased in the country [9,36,37].

The decomposition analysis showed that the estimated difference of using modern contraception methods was 0.022 between BDHS 2014 and BDHS 2018, and it was a significant decrease in using modern contraceptive methods between these two surveys. The shifting due to the change of endowments determinants was 41%. The result showed that if the endowments, the level of explanatory variables remain the same between 2014 and 2018, the usage of modern contraception methods would still have increased by 0.029 due to a change in the effect of explanatory variables. Though the policy makers take various measures to increase the propensity of using modern contraception methods, we observed a shift between two surveys. The Ministry of Health and Family Welfare (MOFHW) of the Government of Bangladesh is implementing Health, Population and Nutrition (HPN) development program to accelerate progress in the areas of health, population, and nutrition so that the use of family planning has increased [3,22]. Globally, the use of modern contraceptives was 55.0% in 2000 which later increased to 57.1% in 2019 [38]. The World Health Organization (WHO) cites limited choice of methods, especially among the young, poor and unmarried, as fear or inexperience, the low quality of services available, and barriers to accessing gender-based services [39].

Among the explanatory variables, respondent’s education (16%) and current working status (35%) significantly effects in narrowing the usage of modern contraception methods between 2014 and 2018. Women’s age had a significant counteracting effect on the increment of using modern contraceptive methods between the two surveys (27%). On the other hand, Women’s opinion on making any decision unveiled as a significant factor for narrowing the gap between two surveys. Women’s opinion on making any decision on their own healthcare had contributed 26% for lessening the shift. Increment of women’s higher education could be uplifted women’s opinion in decision making [5,13,14]. Hasan et al. (2014) concluded that women’s participation on household decision-making empowered them to make decision on using family planning methods [40].

## 5. Strengths and limitations of the study

The main strength of this study lies in its utilization of two consecutive nationally representative surveys, the BDHS 2014 and BDHS 2018, alongside the Blinder-Oaxaca decomposition analysis. By combining these methodologies, the study gains a powerful tool for examining the factors contributing to disparities between two survey periods. Through rigorous analysis of these datasets and methodologies, the study can offer comprehensive insights into the changing landscape of modern contraceptive practices and inform targeted interventions aimed at improving reproductive health outcomes in Bangladesh. However, the study had some limitations. Firstly, since this study was done using secondary data sets, there are many important factors that we could not add due to data limitations. The information of respondent’s family structure was missing in the survey. In addition, whether couple living in a nuclear or joint family or type of family structure was not included. Secondly, we acknowledge that the identification of interaction effects is important for understanding the nuanced relationships between predictors and the outcome variable. However, the large number of categorical variables in our dataset presented a limitation in exploring interaction effects thoroughly. Thirdly, due to the cross-sectional nature of the study, it was not possible to determine the cause-effect relationship.

## 6. Conclusion

The result of this study has several policy implications for Bangladesh. The use of contraceptive methods for proper family planning is considered a significant factor in reducing maternal mortality and morbidity both for child and mother. In this study, we investigated the 2014 and 2018 dataset using Binary logistics regression to identify the influencing factors and Blinder-Oaxaca decomposition techniques to explore the shift between two surveys. The use of modern contraceptive methods has decreased between 2014 and 2018 in Bangladesh. In our study, we identify various socio-demographic factors that influenced the use of modern contraceptive methods over the years. Younger women were more interested in using modern contraception methods than older. The study resulted education as a significant factor in this case. Education will be able to increase the use of modern contraceptive methods among women. The odds of using modern contraceptive methods were high among working women and it increased over the years. The study revealed a shift of the usage of modern contraception methods due to endowments and coefficients components between two surveys. The contribution of endowment factors, women’s age, education, working status, number of living children, and decision variable effects significantly for narrowing the shift between the two surveys. Further research is needed to determine whether any unknown factors are behind this shift.

## References

[1] Islam AZ, Mondal MNI, Khatun ML, Rahman MM, Islam MR, Mostofa MG, et al. Prevalence and Determinants of Contraceptive use among Employed and Unemployed Women in Bangladesh. International Journal of MCH and AIDS. 2016;5: 92. doi: 10.21106/ijma.83 28058196 PMC5187648

[2] Islam MdK, Haque MdR, Hema PS. Regional variations of contraceptive use in Bangladesh: A disaggregate analysis by place of residence. Ali N, editor. PLOS ONE. 2020;15: e0230143. doi: 10.1371/journal.pone.0230143 32210443 PMC7094853

[3] Kibria GMA, Hossen S, Barsha RAA, Sharmeen A, Paul SK, Uddin SMI. Factors affecting contraceptive use among married women of reproductive age in Bangladesh. Journal of Molecular Studies and Medicine Research. 2016;2: 70–79. doi: 10.18801/jmsmr.020116.09

[4] Sachdev HPS. Book: Where Women Have No Doctor: A Health Guide for Women. BMJ. 1998. pp. 1664–1664. doi: 10.1136/bmj.317.7173.1664 9848928 PMC1114457

[5] Andi JR, Wamala R, Ocaya B, Kabagenyi A. Modern contraceptive use among women in Uganda: An analysis of trend and patterns (1995–2011). African Population Studies. 2014;28: 1009. doi: 10.11564/28-0-553 25530666 PMC4269974

[6] Jain R, Muralidhar S. Contraceptive Methods: Needs, Options and Utilization. Journal of Obstetrics and Gynaecology of India. 2011;61: 626–634. doi: 10.1007/s13224-011-0107-7 23204678 PMC3307935

[7] Mohsena M, Kamal N. Determinants of Contraceptive Use in Bangladesh. Ibrahim Medical College Journal. 2016;8: 34–40. doi: 10.3329/imcj.v8i2.26676

[8] Huda FA, Robertson Y, Chowdhuri S, Sarker BK, Reichenbach L, Somrongthong R. Contraceptive practices among married women of reproductive age in Bangladesh: a review of the evidence. Reproductive Health. 2017;14: 1–9. doi: 10.1186/s12978-017-0333-2 28587619 PMC5461624

[9] Ajmal S, Idris A, Ajmal B. Factors affecting contraceptive use and unmet need among currently married women in Afghanistan: further analysis of the 2015 Afghanistan Demographic and Health Survey. Journal of Global Health Reports. 2018;2. doi: 10.29392/joghr.2.e2018037

[10] Degefa Hidru H, Dingeta T, Menigiste B, Etsay B, Gebremedhin H, Berwo M, et al. Modern Contraceptive Utilization and Its Associated Factors among Indigenous and Nonindigenous Married Women of Reproductive Age Group in Jigjiga Town, Eastern Ethiopia, 2018. BioMed Research International. 2020;2020: 1–9. doi: 10.1155/2020/6878075 32596352 PMC7273474

[11] Hossain M, Khan M, Ababneh F, Shaw J. Identifying factors influencing contraceptive use in Bangladesh: evidence from BDHS 2014 data. BMC Public Health. 2018;18: 1–14. doi: 10.1186/s12889-018-5098-1 29378546 PMC5789662

[12] Beson P, Appiah R, Adomah-Afari A. Modern contraceptive use among reproductive-aged women in Ghana: prevalence, predictors, and policy implications. BMC Women’s Health. 2018;18: 1–8. doi: 10.1186/s12905-018-0649-2 30253759 PMC6156857

[13] Das AA. Contraceptive using trends in Bangladesh. International journal of business, social and scientific research. 2015;3: 184–188.

[14] Lakew Y, Reda AA, Tamene H, Benedict S, Deribe K. Geographical variation and factors influencing modern contraceptive use among married women in Ethiopia: evidence from a national population-based survey. Reproductive Health. 2013;10: 1–10. doi: 10.1186/1742-4755-10-52 24067083 PMC3850415

[15] Chintsanya J. Trends and Correlates of Contraceptive Use among Married Women in Malawi: Evidence from 2000–2010 Malawi Demographic and Health Surveys. Calverton, Maryland, USA: ICF International; 2013.

[16] Islam AZ. Factors affecting modern contraceptive use among fecund young women in Bangladesh: does couples’ joint participation in household decision making matter? Reproductive Health. 2018;15: 1–9. doi: 10.1186/s12978-018-0558-8 29929526 PMC6013886

[17] Islam AZ, Rahman M, Mostofa MdG. Association between contraceptive use and socio-demographic factors of young fecund women in Bangladesh. Sexual & Reproductive Healthcare. 2017;13: 1–7. doi: 10.1016/j.srhc.2017.05.001 28844349

[18] Habyarimana F, Ramroop S. Spatial Analysis of Socio-Economic and Demographic Factors Associated with Contraceptive Use among Women of Childbearing Age in Rwanda. International Journal of Environmental Research and Public Health. 2018;15: 2383. doi: 10.3390/ijerph15112383 30373248 PMC6265926

[19] Alo OD, Daini BO, Omisile OK, Ubah EJ, Adelusi OE, Idoko-Asuelimhen O. Factors influencing the use of modern contraceptive in Nigeria: a multilevel logistic analysis using linked data from performance monitoring and accountability 2020. BMC Women’s Health. 2020;20: 1–9. doi: 10.1186/s12905-020-01059-6 32883259 PMC7650288

[20] Lwelamira J, Mnyamagola G, Msaki MM. Knowledge, Attitude and Practice (KAP) Towards Modern Contraceptives Among Married Women of Reproductive Age in Mpwapwa District, Central Tanzania. Current Research Journal of Social Sciences. 2012;4: 235–245.

[21] National Institute of Population Research and Training (NIPORT), Mitra and Associates, Macro International. Bangladesh Demographic and Health Survey 2007. Dhaka, Bangladesh and Calverton, Maryland, USA; 2007.

[22] National Institute of Population Research and Training (NIPORT), Mitra Associates, ICF International. Bangladesh Demographic and Health Survey 2011. Dhaka, Bangladesh and Calverton, Maryland, USA; 2011.

[23] National Institute of Population Research and Training (NIPORT), Mitra and Associates, ICF International. Bangladesh Demographic and Health Survey 2014. Dhaka, Bangladesh and Calverton, Maryland, USA; 2014.

[24] National Institute of Population Research and Training (NIPORT), Mitra and Associates, ICF International. Bangladesh Demographic and Health Survey 2017–18. Dhaka, Bangladesh and Calverton, Maryland, USA; 2018.

[25] Aviisah PA, Dery S, Atsu BK, Yawson A, Alotaibi RM, Rezk HR, et al. Modern contraceptive use among women of reproductive age in Ghana: analysis of the 2003–2014 Ghana Demographic and Health Surveys. BMC Women’s Health. 2018;18. doi: 10.1186/s12905-018-0634-9 30126389 PMC6102847

[26] Sidibé S, Delamou A, Camara BS, Dioubaté N, Manet H, El Ayadi AM, et al. Trends in contraceptive use, unmet need and associated factors of modern contraceptive use among urban adolescents and young women in Guinea. BMC Public Health. 2020;20: 1–10. doi: 10.1186/s12889-020-09957-y 33261605 PMC7706031

[27] Feyisso M, Belachew T, Tesfay A, Addisu Y. Differentials of modern contraceptive methods use by food security status among married women of reproductive age in Wolaita Zone, South Ethiopia. Archives of Public Health. 2015;73. doi: 10.1186/s13690-015-0089-5 26753092 PMC4705916

[28] Belda SS, Haile MT, Melku AT, Tololu AK. Modern contraceptive utilization and associated factors among married pastoralist women in Bale eco-region, Bale Zone, South East Ethiopia. BMC Health Services Research. 2017;17. doi: 10.1186/s12913-017-2115-5 28288616 PMC5348813

[29] Islam S, Islam Ma, Padmadas SS. High Fertility Regions in Bangladesh: A Marriage Cohort Analysis. Journal of Biosocial Science. 2010;42: 705–719. doi: 10.1017/S0021932010000428 20868540

[30] Goni A, Rahman M. The impact of education and media on contraceptive use in Bangladesh: A multivariate analysis. International Journal of Nursing Practice. 2012;18: 565–573. doi: 10.1111/ijn.12013 23181958

[31] Mustafa R, Afreen U, Hashmi HA. Contraceptive knowledge, attitude and practice among rural women. Journal of the College of Physicians and Surgeons—Pakistan. 2008;18: 542–545. 18803890

[32] Senarath U, Nalika Sepali Gunawardena. Women’s Autonomy in Decision Making for Health Care in South Asia. Asia Pacific Journal of Public Health. 2009;21: 137–143. doi: 10.1177/1010539509331590 19190000

[33] Sserwanja Q, Turimumahoro P, Nuwabaine L, Kamara K, Musaba MW. Association between exposure to family planning messages on different mass media channels and the utilization of modern contraceptives among young women in Sierra Leone: insights from the 2019 Sierra Leone Demographic Health Survey. BMC Women’s Health. 2022;22. doi: 10.1186/s12905-022-01974-w 36114503 PMC9479264

[34] Nsanya MK, Atchison CJ, Bottomley C, Doyle AM, Kapiga SH. Modern contraceptive use among sexually active women aged 15–19 years in North-Western Tanzania: results from the Adolescent 360 (A360) baseline survey. BMJ Open. 2019;9: e030485. doi: 10.1136/bmjopen-2019-030485 31467055 PMC6720144

[35] Casey SE, Gallagher MC, Kakesa J, Kalyanpur A, Muselemu J-B, Rafanoharana RV, et al. Contraceptive use among adolescent and young women in North and South Kivu, Democratic Republic of the Congo: A cross-sectional population-based survey. Wickramage K, editor. PLOS Medicine. 2020;17: e1003086. doi: 10.1371/journal.pmed.1003086 32231356 PMC7108687

[36] Osmani AK, Reyer JA, Osmani AR, Hamajima N. Factors influencing contraceptive use among women in Afghanistan: secondary analysis of Afghanistan Health Survey 2012. Nagoya J Med Sci. 2015;77: 551–561. 26663934 PMC4664587

[37] Aslam SK, Zaheer S, Qureshi MS, Aslam SN, Shafique K. Socio-Economic Disparities in Use of Family Planning Methods among Pakistani Women: Findings from Pakistan Demographic and Health Surveys. PLOS ONE. 2016;11. doi: 10.1371/journal.pone.0153313 27055164 PMC4824490

[38] World Health Organization. World Health Organization. In: Who.int [Internet]. World Health Organization; 2022. Available: https://www.who.int/.

[39] Collins FS, Aminu MA. Barriers to adoption of modern contraceptive methods among women of reproductive age in northern Nigeria. International Journal of Management, Social Sciences. 2021;4: 301–311.

[40] Hasan MS, Dana GPT, Jafrin N. Determinants of Contraceptive Use in Rural Bangladesh. Journal of Population and Development. 2014;1: 53–72.

